# Immunocytological analysis of meiotic recombination in two anole lizards (Squamata, Dactyloidae)

**DOI:** 10.3897/CompCytogen.v11i1.10916

**Published:** 2017-03-06

**Authors:** Artem P. Lisachov, Vladimir A. Trifonov, Massimo Giovannotti, Malcolm A. Ferguson-Smith, Pavel M. Borodin

**Affiliations:** 1 Institute of Cytology and Genetics, Russian Academy of Sciences, Siberian Branch, Novosibirsk 630090, Russia; 2 Institute of Molecular and Cellular Biology, Russian Academy of Sciences, Siberian Branch, Novosibirsk 630090, Russia; 3 Novosibirsk State University, Novosibirsk 630090, Russia; 4 Dipartimento di Scienze della Vita e dell’Ambiente, Università Politecnica delle Marche, via Brecce Bianche, 60131 Ancona, Italy; 5 Cambridge Resource Centre for Comparative Genomics, Department of Veterinary Medicine, University of Cambridge, Madingley Road, Cambridge CB3 0ES, UK

**Keywords:** Synaptonemal complex, chromosomes, crossing over, *Anolis*, *Deiroptyx*, lizard, Reptilia

## Abstract

Although the evolutionary importance of meiotic recombination is not disputed, the significance of interspecies differences in the recombination rates and recombination landscapes remains under-appreciated. Recombination rates and distribution of chiasmata have been examined cytologically in many mammalian species, whereas data on other vertebrates are scarce. Immunolocalization of the protein of the synaptonemal complex (SYCP3), centromere proteins and the mismatch-repair protein MLH1 was used, which is associated with the most common type of recombination nodules, to analyze the pattern of meiotic recombination in the male of two species of iguanian lizards, *Anolis carolinensis* Voigt, 1832 and *Deiroptyx coelestinus* (Cope, 1862). These species are separated by a relatively long evolutionary history although they retain the ancestral iguanian karyotype. In both species similar and extremely uneven distributions of MLH1 foci along the macrochromosome bivalents were detected: approximately 90% of crossovers were located at the distal 20% of the chromosome arm length. Almost total suppression of recombination in the intermediate and proximal regions of the chromosome arms contradicts the hypothesis that “homogenous recombination” is responsible for the low variation in GC content across the anole genome. It also leads to strong linkage disequilibrium between the genes located in these regions, which may benefit conservation of co-adaptive gene arrays responsible for the ecological adaptations of the anoles.

## Introduction

Meiotic recombination (crossing over) plays a dual role in sexually reproducing organisms. At least one crossover per chromosome is necessary and sufficient to secure orderly segregation of homologous chromosomes during the first meiotic division. Crossing over shuffles allele combinations between homologous chromosomes, increasing the genetic variation in the progeny, on the one hand, and shaping local patterns of GC-content (i.e., creating or modifying isochores) along the chromosome length, on the other hand (Eyre-Walker and Hurst 2001).

The number and distribution of the crossovers along a chromosome depends on its length, chromatin composition, genetic content and crossover interference (Lynn et al. 2002, Pardo-Manuel de Villena and Sapienza 2001). The longer the chromosome, the more crossovers it may accommodate. Euchromatic regions show higher recombination rate than heterochromatic regions. At the DNA sequence level, recombination rate at hotspots can be hundreds of times higher than in the adjacent regions (Zickler and Kleckner 2016). The occurrence of a crossover usually reduces the probability of another crossover close by. This phenomenon, which is called crossover interference, also makes a substantial contribution to the number and distribution of crossovers along the chromosome (Moens 2006).

The patterns of crossover distribution have been studied across a variety of vertebrates such as fish (Moens 2006, Lisachov et al. 2015), birds (Pigozzi 2001, Calderon and Pigozzi 2006) and mammals (Anderson et al. 1999, Borodin et al. 2008, Basheva et al. 2008). Usually vertebrate chromosomes show an uneven crossover pattern with more or less pronounced recombination hotspots and low-recombining regions.

There are several hypotheses which connect the recombination landscape with species’ ecology and speciation (Barton and Otto 2005, Ortiz-Barrientos et al. 2016). Lower numbers of crossovers and their uneven distribution, which creates recombination “valleys”, is thought to be beneficial for preserving adaptive allele combinations in ecologically specialized species living in stable conditions, and for suppressing interspecies gene flow in hybrid zones. Higher recombination, which creates more diverse offspring, is beneficial in unstable and diverse conditions (Burt and Bell 1987, Otto and Barton 2001).

Reptiles are particularly interesting organisms in which to study the evolution of recombination because they show a wide array of karyotypes and ecological specializations, and extensive homology and synteny between reptilian and avian chromosomes has been demonstrated (Pokorná et al. 2012). Data on recombination patterns in reptiles remain scarce. Most studies used chiasma counts and distribution at diakinesis-metaphase I (Cobror et al. 1986, Lamborot et al. 2012, Reed et al. 1992). However, the resolution of chiasmata analysis is rather poor, because the chromosomes at metaphase I are condensed, making precise chiasmata localization along the chromosome difficult. Recently recombination in one reptile species (*Crocodylus porosus* Schneider, 1801) was estimated via linkage analysis of microsatellite markers (Miles et al. 2009). However, the resolution of linkage analysis depends on the number and distribution of available markers. Low density of mapping leads to underestimation of the recombination rate.

The most widespread technique for studying recombination rate and localization is by immunofluorescent mapping of MLH1 (the mismatch repair protein associated with mature recombination nodules) along the synaptonemal complexes (SCs) at prophase (Moens 2006, Pigozzi 2001, Borodin et al. 2008). MLH1 marks about 90–95 % of all recombination events in mouse (Guillon et al. 2005), thus providing reliable estimates of the total recombination rate, as well as the frequency and distribution of recombination events in individual chromosomes (Froenicke et al. 2002).

One of the most species-rich and diverse reptilian clades are iguanians (infraorder Iguania), which include nearly 30% of all lizard species (Uetz and Hošek 2005). Iguanians are further subdivided into pleurodonts (Pleurodonta) and acrodonts (Acrodonta). The former clade includes New World and Madagascan species (former family Iguanidae *sensu lato*), and the latter includes chameleons (Chamaeleonidae Rafinesque, 1815) and Old World and Australian dragon lizards (Agamidae Gray, 1827). Many of them have a conservative karyotype with 2n = 36, including 12 submetacentric and metacentric macrochromosomes and 24 microchromosomes. This karyotype is presumed to be ancestral for Iguania (Deakin et al. 2016).

Among iguanians, anoles (Dactyloidae Fitzinger, 1843, Pleurodonta) are one of the best studied lineages. They are the classical model organisms in studies of reptilian ecology, evolution, biogeography, karyology and genetics (Hertz et al. 2013, Fleishman and Pallus 2010, Giovannotti et al. 2016). One of their representative, *Anolis carolinensis* Voigt, 1832, is the first reptile whose genome was almost fully sequenced (Alföldi et al. 2011).

In this study, we assessed the pattern of meiotic recombination in two anole species, *Anolis carolinensis* and *Deiroptyx coelestinus* (Cope, 1862). Although these species are separated by a relatively long evolutionary history (Nicholson et al. 2012), they both possess the ancestral iguanian karyotype (Gorman 1973). We examined the number and distribution of crossovers along their macrochromosomes using immunofluorescent localization of MLH1 at SC spreads.

## Materials and methods

### Specimens

The specimens, two male *Anolis carolinensis* and one male *Deiroptyx coelestinus*, were purchased from commercial breeders. Handling and euthanasia of the animals were performed according to the protocols approved by the Animal Care and Use Committee at the Institute of Cytology and Genetics. The specimens were deposited in the research collections of the institute.

### Chromosome preparation and immunostaining

The spreads of meiotic cells were prepared according to the protocol of Peters et al. (1997). Immunostaining was performed according to the protocol described by Anderson et al. (1999) using rabbit polyclonal anti-SYCP3 (1:500, Abcam), mouse monoclonal anti-MLH1 (1:50, Abcam), and human anticentromere (ACA) (1:100, Antibodies Inc) primary antibodies. As secondary antibodies Cy3-conjugated goat anti-rabbit (1:500, Jackson ImmunoResearch), FITC-conjugated goat anti-mouse (1:50, Jackson ImmunoResearch), FITC-conjugated donkey anti-human (1:100, Vector Laboratories) were used. All antibodies were diluted in PBT (3% bovine serum albumin and 0.05% Tween 20 in 1xPBS). A solution of 10% PBT was used for blocking non-specific antibody binding. Primary antibody incubation was performed overnight in a humid chamber at 37°C, and secondary antibody incubation was performed for 1 h at 37°C. Finally, slides were mounted in Vectashield with DAPI (Vector Laboratories) to stain DNA and reduce ﬂuorescence fading. After image acquisition of the immunofluorescent signals, the slides were subjected to FISH.

The preparations were visualized with an Axioplan 2 Imaging microscope (Carl Zeiss) equipped with a CCD camera (CV M300, JAI), CHROMA filter sets, and ISIS4 image processing package (MetaSystems GmbH).

### Image processing and analysis

Brightness and contrast of all images were enhanced using Corel PaintShop Photo Pro X6 (Corel Corp). The centromeres were identified by the ACA foci. The MLH1 signals were scored only if they were localized on SCs. The length of the SC of each chromosome arm was measured in micrometers and the positions of centromeres and MLH1 foci in relation to the centromeres were recorded using MicroMeasure 3.3 software (Reeves 2001). Relative distances between the MLH1 foci and between the MLH1 foci and centromeres were calculated as fractions of the SC and arm length respectively.

To map the MLH1 foci distribution along the macroSCs we calculated the absolute position of each MLH1 focus multiplying the relative position of each focus by the average absolute length for the corresponding chromosome arm. These data were pooled for each arm and plotted to represent a recombination map.

Statistica 6.0 software package (StatSoft) was used for descriptive statistics. MLH1 foci distribution along the SCs was analyzed using CODA v.1.1 software (Gauthier et al. 2011). To estimate the strength of crossover interference we used the shape parameter (ν) of the gamma distribution. This distribution describes the probability of the distances between MLH1 foci under the assumption that their precursors are randomly placed along the bivalent and every *v*-th precursor would result in a focus. The ν-value varies from 1 (every precursor results in a focus, i.e. no interference) to 20 (high interference).

## Results

**Figure 1.**
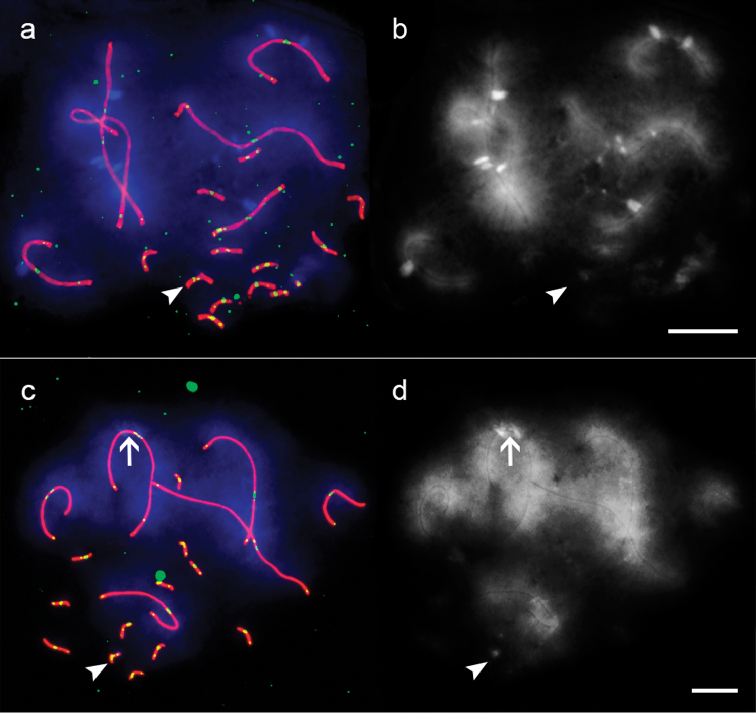
The SC spreads of *Anolis carolinensis* (**a, b**) and *Deiroptyx coelestinus* (**c, d**). **a, c** immunofluorescence and DAPI. Red: SYCP3, green: centromere and MLH1, blue: DAPI **b, d** DAPI channel separately. Arrowheads show the XY bivalent (Lisachov et al. in press). Arrow shows the DAPI+ band on the SC2 of *Deiroptyx coelestinus* . Scales bars: 5 µm.

Figure 1 shows the microphotographs of the surface spreads of the pachytene spermatocyte nuclei of *Anolis carolinensis* and *Deiroptyx coelestinus*. Each SC spread contains 6 macroSCs and 12 microSCs (2n = 36). We analyzed 96 pachytene nuclei of *Anolis carolinensis* and 100 nuclei of *Deiroptyx coelestinus*

All the macroSCs of *Anolis carolinensis* show DAPI-positive bands in their pericentromeric regions. In *Deiroptyx coelestinus* such bands were detected only at SC2 and at one microSC. Such bands are observed in many species and generally correspond to C-heterochromatin. They mainly contain satellite repeats (Charlesworth et al. 1994). The centromeric indices of the macrochromosomes of both species were around 0.45–0.5, except for chromosome 3 in *Deiroptyx coelestinus* which had average centromeric index of 0.35. Thus in *Deiroptyx coelestinus*, we were able to identify SC2 by its DAPI-positive band and SC3 by its centromeric index. The SCs 1, 4, 5, and 6 of *Deiroptyx coelestinus* and all macroSC of *Anolis carolinensis* were identified by their length (Table 1).

The mean number of MLH1 foci on each of the macrochromosomal bivalents was calculated (Table 1). We used only the SCs which contained at least one MLH1 focus.

**Table 1.** Average SC length (µm) and of MLH1 foci number (±S.D.) in macroSCs in two anole species.

	*Anolis carolinensis*		*Deiroptyx coelestinus*	
SC rank	SC length (µm)	No. of MLH1 foci	SC length (µm)	No. of MLH1 foci
1	28.8±5.1	1.90±0.47	25.7±5.2	1.98±0.34
2	25.5±4.3	1.88±0.45	24.5±4.7	1.90±0.30
3	20.1±3.0	1.92±0.43	18.5±3.6	1.69±0.53
4	18.0±2.7	1.89±0.40	16.9±2.9	1.68±0.51
5	14.3±2.0	1.80±0.43	12.6±2.1	1.34±0.48
6	10.9±1.3	1.45±0.50	10.2±1.8	1.11±0.31

**Figure 2.**
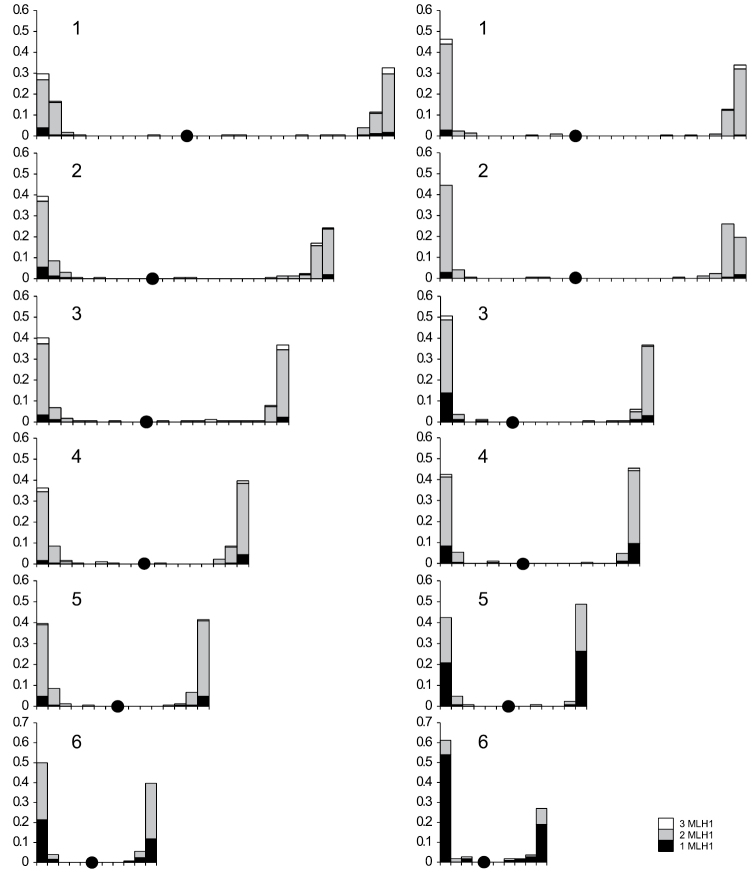
The distribution of MLH1 foci along the macrochromosomes of *Anolis carolinensis* and *Deiroptyx coelestinus*. The X-axis shows the position of MLH1 foci, the marks on this axis are separated by 1 μm. Black dots indicate centromeres. The Y-axis indicates the frequency of MLH1 foci in each 1 μm – interval. Stacked columns show the frequency for the SCs containing MLH1 foci at each interval.

The distribution of MLH1 foci along all the macroSCs in both species was extremely uneven (Fig. 2). The prominent peaks of the foci occurred near the telomeres of both arms, and the distal 20% of the arm length contained more than 90% of all foci. The bivalents with MLH1 foci located beyond the terminal regions usually carried three foci, and two of them were always located near the telomeres. Thus, the intermediate MLH1 foci may be considered as the second crossovers pushed proximally by crossover interference (Fig. 2). We detected a moderate interference in anoles. In *Anolis carolinensis* the ν-value was estimated as 5.6 (95% CI 5.0–6.7), and in *Deiroptyx coelestinus* it was estimated as 5.0 (95% CI 4.5–5.5).

## Discussion

The most interesting feature of the recombination pattern of *Anolis carolinensis* and *Deiroptyx coelestinus* macrochromosomes is an extreme polarization of the recombination events. Similar terminal localization of crossovers was previously observed in several anole species, including *Anolis carolinensis*, using chiasmata analysis at diakinesis-metaphase I (Gorman and Atkins 1968, Gorman and Atkins 1966, Beçak et al. 1964).

Subtelomeric peaks in the distribution of crossovers are common for most vertebrates. This phenomenon is explained by the fact that meiotic pairing of the homologs is usually initiated at the telomeres (Zickler and Kleckner 2016). Whereas this general pattern is common for most species, the numbers of crossovers and their exact distribution varies greatly between taxa. However, such an extreme pattern of crossovers, which we observe in the anoles, is unusual.

Crossover interference is unlikely to be the cause of almost complete suppression of recombination beyond the subtelomeric regions, because the macrochromosomes of both species demonstrate a rather moderate degree of interference. Our estimate of the ν-value (approximately 5.0) is the first estimate of this parameter in reptiles, so we can only compare it with estimates obtained for mammalian chromosomes of similar size. It was substantially lower than the values detected in the largest chromosomes of common shrews (11.1: Borodin et al. 2008) and mouse (13.7‒14.4: De Boer et al. 2006).

Additional evidence against crossover interference as the cause of the extreme distal location of the crossovers is the fact that single crossovers are also located distally. Single crossovers tend to be located in the middle of mammalian chromosomes, because they suppress the occurrence of other crossovers at both chromosome ends (Anderson et al. 1999, Borodin et al. 2008). The distribution observed in the anole macrochromosomes may be determined by a very early and short time window for the initiation of homologous pairing and recombination.

Almost total suppression of recombination in the intermediate and proximal regions of chromosome arms would lead to strong linkage disequilibrium between the genes located in these regions. This may benefit the conservation of co-adaptive gene arrays or “supergenes” (Pál and Hurst 2003, Thompson and Jiggins 2014, Charlesworth 2016). According to the Red Queen theory, low recombination is favored under stable environmental conditions and stabilizing selection (Otto and Michalakis 1998). Indeed, the ecology and morphology of anoles have remained unchanged for tens of millions of years, which is supported by molecular phylogeny (Nicholson et al. 2012) and also by the remarkable finds of fossil anoles preserved in the Dominican amber (Sherratt et al. 2015). Perhaps, the recombination suppression serves to keep the “supergenes” which are responsible for their lifestyle adaptations.

The divergence between *Anolis* and *Deiroptyx* is one of the basal radiations among Dactyloidae (Nicholson et al. 2012). Therefore, this recombination pattern is probably ancestral for the whole family. It remains unknown if a similar recombination pattern is conserved in the anole lineages (e. g. *Norops* Wagler, 1830, *Ctenonotus* Fitzinger, 1843) which underwent a series of chromosome fusions and fissions (2n = 28–30, 2n = 40 in comparison with the ancestral 2n = 36), which led to the appearance of new macrochromosomes (Castiglia et al. 2013).

The results of our analysis of crossover distribution along anole macrochromosomes might shed light on a peculiarity of their genome organization. One of the specific characters of the genomes of cold-blooded vertebrates is weak regional variation in GC-content (i.e. less prominent isochore structure) in comparison with birds and mammals (Costantini et al. 2009). Until recently (Figuet et al. 2014, Costantini et al. 2016) it was even thought (Fujita et al. 2011, Alföldi et al. 2011) that in the genome of *Anolis carolinensis* there is no isochore structure at all. In mammals, GC-rich isochores are known to be located at recombination hotspots, and it is suggested that they are formed by recombination via GC-biased gene conversion (gBGC) (Eyre-Walker and Hurst 2001). Considering this fact, Olmo (2008) postulated that the relatively homogenous distribution of the GC-content in reptiles probably reflects the relatively homogenous distribution of crossovers along reptilian chromosomes. According to Olmo, this should produce more points for chromosomal rearrangements (since they originate as recombination errors), which should reinforce karyotypic evolution and therefore speciation.

The extremely distal localization of crossovers in the males of both anole species here analyzed might be considered as evidence against this hypothesis. The weak prominence of isochores in reptiles is apparently produced by some forces other than gBGC and does not reflect the distribution of recombination hotspots. Moreover, intense and homogenous recombination, which is known for example for birds, is apparently not enough to drive intense karyotypic evolution, since bird karyotypes are the most conservative and archaic among all vertebrates (Uno et al. 2012).

There are two additional important points to be considered in the discussion. In some reptile species chiasma number and localization depend on environmental conditions (Cobror et al. 1986, King and Hayman 1978). It remains possible that the median regions of the anole chromosomes could recombine in other conditions. However, we consider this possibility unlikely since the chiasma distributions found in previous studies in anoles from wild populations agree with the MLH1 distribution found in our study (Gorman and Atkins 1968, Gorman and Atkins 1966, Beçak et al. 1964). Further studies of recombination under alternative controlled conditions are necessary to clarify this point.

Sex difference in recombination rate and distribution should also be taken into account. Females tend to have higher recombination rates than males and more even distribution of crossovers along the chromosomes (Burt et al. 1991, Mank 2009). We cannot exclude that the median regions recombine in female meiosis of anoles. In mammals and birds, newborn females or even female embryos are used to obtain the female SC spreads. We did not detect any meiotic divisions in the gonads of newborn and juvenile lizards. In our opinion, lampbrush chromosome analysis may help to solve this question.

## Conclusions

For the first time we directly assessed meiotic recombination in reptilian species using MLH1 mapping in SCs. We found that, in male anole lizards *Anolis carolinensis* and *Deiroptyx coelestinus*, MLH1 foci are mainly located in the terminal parts of the chromosome arms, whereas recombination intensity in the median parts of the chromosomes is extremely low. This result disagrees with the hypothesis of “homogenous recombination” as the cause of low isochore prominence in the genome of anoles. However, recombination in females has to be studied before drawing any final conclusions about overall recombination rate and distribution in anoles.

## Competing interests

The authors declare that they have no competing interests.
